# The Antitoxin Treatment of Diphtheria in Buffalo1Read at the twenty-ninth annual meeting of the Medical Association of Central New York, at Rochester, October 20, 1896.

**Published:** 1897-05

**Authors:** Irving M. Snow

**Affiliations:** Buffalo, N. Y.; Clinical professor of diseases of children, University of Buffalo; member American Pediatric Society; physician to Buffalo Fresh Air Mission Hospital, etc. 476 Franklin Street


BUFFALO MEDICAL JOURNAL.

VOL. XXXVI. MAY, 1897. NO. 10.

### Original Communications.

### THE ANTITOXIN TREATMENT OF DIPHTHERIA IN BUFFALO.[1]

BY IRVING M. SNOW, M. D., Buffalo, N. Y.,
Clinical professor of diseases of children, University of Buffalo; member American Pediatric Society; physician to Buffalo Fresh Air Mission Hospital, etc.

THE paper which I am to read to you today deals with the results of the antitoxin treatment of diphtheria in private practice in Buffalo. I was asked by the collective investigations committee of the American Pediatric Society to gather local statistics of the serum treatment of diphtheria. From the circulars of the society I have compiled some interesting statistics, and I am able to show you what the results of the serum treatment of diphtheria are in Buffalo, how our therapeutic success compares with other cities and lastly the effect of the serum treatment in this city upon the general mortality from membranous and diphtheritic croup and diphtheria.

The American Pediatric Society was of the opinion that a much clearer idea of the value of the serum treatment might be obtained by collecting cases seen in private practice—among strong, well-nourished individuals, surrounded by efficient nursing and prompt medical attention—than by massing hospital statistics together, where the subjects are drawn from the poorest and most physically degenerate of our population. After persistent inquiry, I learned that about 300 cases had had the advantage of the serum treatment in private practice in Buffalo up to May 1, 1896. As there annually occurs in this city between 600 and 700 cases of diphtheritic disease I would conclude that in the year ending May 1, 1896, from 35 to 40 per cent. were treated by antitoxin injections. Of some 250

1. Read at the twenty-ninth annual meeting of the Medical Association of Central New York, at Rochester, October 20, 1896.

circulars sent out, only 164 were returned to me correctly filled. Doubtless many interesting cases were omitted from the carelessness or physical indolence of physicians not filling out the blanks.

Among my 164 cases there occurred 29 deaths, a mortality of 17.7 per cent. In only 108 was the diagnosis confirmed by culture. These cases show a mortality of 13 per cent. Of the 56 cases diagnosticated clinically, 15 or 26.6 per cent. died. As the group of cases diagnosticated clinically were far more fatal, as most of them were clearly diphtheria from the description of the location of the membrane and the general symptomatology, I am inclined to accept the entire number of cases as being caused by the Klebs-Löffler bacillus. This mortality of 17.7 per cent. compares very unfavorably with the general statistics of the American Pediatric Society, whose 3,384 cases show a death-rate of only 13 per cent. That their cases were equally severe, or even more malignant, is shown by the fact that only 12.2 per cent. of the Buffalo cases received operative treatment, while 16.7 per cent. of the American Pediatric Society cases were tracheotomised or intubated.

If cases dying within 24 hours after injection be excluded, the American Pediatric Society's returns show a mortality of 8.8 per cent. Our lack of success with the Heil serum might be due (1) to unusual malignancy, which I have shown is not the case by the lower percentage of cases requiring operative treatment in Buffalo ; (2) to the injections being given too late, which I also doubt, as 19 out of 28 fatal cases were treated before the close of the 3d day—1st day, 4; 2d day, 8 ; 3d day, 7 ; 4th day, 2 ; after 5 days, 7 ; (3) to poor serum. The serum used was Behring's, Gronson's or Gibier, all of undoubted purity ; (4) insufficient dose, which I am inclined to believe, although the number of antitoxin units given was seldom mentioned.

Comparing the local death-rate, according to age, the local death-rate is always higher.

| Years .......... | 0–2 | 2–5 | 5–10 | 10–15 | 15–20 | 20 |
|---|---|---|---|---|---|---|
| American Pediatric Society.. | 21.7 per ct. | 13.7 per ct. | 12.2 per ct. | 6.8 per ct. | 3.6 per ct. | 4.2 per ct. |
| Buffalo.......... | 33.3 per ct. | 18.5 per ct. | 16.2 per ct. | 14.3 per ct. | 0.7 per ct. | 9.5 per ct. |

The results of intubation with serum treatment are also not as good in Buffalo. Of 18 intubations 6-33.3 died ; of 533 cases of intubation with serum treatment from the American Pediatric Society's returns, 25.9 per cent died. Our Buffalo statistics are, however, identical with those of O'Dwyer and McNaughton, who also lost one-third of their cases, intubated under the new treatment. Yet how noticeable is the advance in therapeutics, when before the serum treatment from 66 to 73 per cent. of cases of diphtheritic laryngitis intubated died. Indeed, the new serum treatment has shown itself to be of extraordinary efficiency in preventing or alleviating diphtheritic laryngeal stenoses.

Of the cases that recovered 8 or 5.9 per cent. suffered from paralysis or neuritis. As the society statistics showed paralytic sequelæ in 9.4 per cent., we may conclude that many cases of post diphtheritic paralysis were not recorded or were overlooked by the physicians making my returns.

Lennoxe Brown, out of 1,000 cases of diphtheria, found that neuritis or paralysis was present in 14 per cent. Sannê reports 11 per cent. Yet the general opinion is that the new serum treatment of diphtheria will slightly increase the proportion of paralytic sequelæ as many desperate cases survive.

An interesting feature of the new therapeutics is the number of patients who die of late cardiac paralysis; doubtless many would have succumbed earlier from suffocation or from diphtheritic toxemia.

Of my fatal cases, 8 died of sepsis, 8 of cardiac failure, 1 of pneumonia, and 1 of nephritis existing before the serum was injected. No case of nephritis or of sudden death followed the antitoxin injections. Among the 135 cases of diphtheria that survived were 3 cases of pneumonia, 3 of nephritis, 6 of sepsis, 2 of heart failure.

Broncho-pneumonia, so fatal a complication of diphtheria, is strikingly absent from the new statistics. Possibly the rarity of broncho-pneumonia, commonly due to a secondary injection of the streptococcus pyogenes, may be owing to the fact that the curative value of the serum is especially great in laryngeal diphtheria. The remedy lessens the frequency and severity of the laryngeal inflammation. The larynx is the principal avenue of infection in diphtheritic broncho-pneumonia.

It would be interesting to compare the results of the antitoxin method with those treated by the older methods in this city. Unfortunately there is no date available for this purpose. In the District of Columbia, Dr. Samuel S. Adams was able to show a noticeable difference in favor of antitoxin.

One hundred and thirty-five cases were treated without serum, average age, 5.96 years ; deaths, 34.8 per cent ; 176 cases treated with antitoxin injections, average age, 6.51, percentage of deaths, 14.77 per cent., a difference in favor of antitoxin of 20.04 per cent.

Has the extensive use of antitoxin actually lessened the gross number of deaths from diphtheritic diseases in Buffalo ? No.

| | Cases of Diphtheria Reported. | Deaths from Diphtheria—Membranous and Diphtheritic Croup. |
|---|---|---|
| First 6 Months, 1894 | 173 | 60 |
| " 1895 | 313 | 90 |
| " 1896 | 419 | 104 |

During this period the general death-rate has steadily diminished.

| | Deaths from Respiratory Disease. |
|---|---|
| First 6 Months of 1894 | 404 |
| " 1895 | 286 |
| " 1896 | 354 |

| | | Proportion from Diphtheritic Disease. |
|---|---|---|
| Total number of deaths first half, 1894 | 2,478 | 2.4 per cent. |
| " " " 1895 | 2,295 | 3.9 " |
| " " " 1896 | 1,960 | 5.3 " |

There has, therefore, been not only a larger number of deaths from diphtheritic disease during the period quoted, but the proportion of deaths caused by the Klebs-Löffler bacillus is increasingly great. This, I believe, is due to the steady spread of the disease throughout the city, to its assuming an endemic character and not to the failure of the antitoxin treatment.

The average mortality from diphtheria in American cities was from 30 to 40 per cent. The serum treatment has reduced it to 17.7 in Buffalo, and to 13 per cent. for the majority of American communities. Again, consider the very favorable showing of our intubation cases, 66.6 per cent. of recoveries from the worst type of diphtheria. Most of the physicians (40) returning the committee blanks were enthusiastic in praise of the curative value of the Heil serum. In 21 of the 135 recoveries, the physicians declare that the remedy was the means of saving life.

It is my opinion that at least in children the dose of antitoxin injected is usually too small. The advice of the committee of the American Pediatric Society is to give the serum early in a clinical diagnosis and not to wait for a bacteriological culture.

For a child over two years with laryngeal stenosis, or with threatening symptoms, 1,500 to 2,000 units for the first injection, to be repeated in 18 to 24 hours, if no improvement ; a third dose after a similar interval if necessary.

For severe cases in children under two and mild ones over two, 1,000 units, repeat if necessary; second dose generally not required. Dosage to be estimated in antitoxin units and not in amount of serum.

476 Franklin Street.

